# Crystal Structure of the Pyrazinamidase of *Mycobacterium tuberculosis*: Insights into Natural and Acquired Resistance to Pyrazinamide

**DOI:** 10.1371/journal.pone.0015785

**Published:** 2011-01-24

**Authors:** Stéphanie Petrella, Nathalie Gelus-Ziental, Arnaud Maudry, Caroline Laurans, Rachid Boudjelloul, Wladimir Sougakoff

**Affiliations:** 1 UPMC Université Paris 6, ER 5 (EA1541) Bactériologie-Hygiène, Faculté de Médecine Pitié-Salpêtrière, Paris, France; 2 INSERM, U908 et FRE3249 CNRS, Université Lille I, Villeneuve d'Ascq, France; 3 Equipe Opérationnelle d'Hygiène et d'Infectiologie, Centre Hospitalier Victor Provo, Roubaix, France; 4 INSERM, UMRS-872-12 Pitié-Salpêtrière, LRMA, Paris, France; University of Cambridge, United Kingdom

## Abstract

Pyrazinamidase (PncA) activates the first-line antituberculous drug pyrazinamide into pyrazinoic acid. The crystal structure of the *Mycobacterium tuberculosis* PncA protein has been determined, showing significant differences in the substrate binding cavity when compared to the pyrazinamidases from *Pyrococcus horikoshii* and *Acinetobacter baumanii*. In *M. tuberculosis*, this region was found to hold a Fe^2+^ ion coordinated by one aspartate and three histidines, one of them corresponding to His57 which is replaced by Asp in *Mycobacterium bovis*, a species naturally resistant to pyrazinamide. The binding cavity also contains a Cys138-Asp8-Lys96 motif evocating a cysteine-based catalytic mechanism. Mutants have been constructed and investigated by kinetic and thermal shift assays, highlighting the importance of protein folding and thermal stability in the pyrazinamidase activity.

## Introduction

Pyrazinamide (PZA), which is an analogue of nicotinamide, is an important first-line drug used in the short-course treatment of tuberculosis. This antibiotic plays a key role in shortening the duration of antituberculous treatment because of its activity against the persisting tubercle bacilli at an acidic pH [1]. PZA, which is a prodrug devoid of significant antibacterial activity, is metabolized into its active form, pyrazinoic acid (POA), by the amidase activity of the *Mycobacterium tuberculosis* nicotinamidase/pyrazinamidase [2] (referred to as MtPncA), encoded by the *pncA* gene [3].

Mutations in *pncA* represent the major mechanism of PZA resistance in *M*. *tuberculosis* [3]–[8]. One of the most striking features of the mutations reported in *pncA* from clinical isolates resistant to PZA is their diversity, with hundreds of mutations scattered throughout the *pncA* gene, including missense mutations, insertions and deletions [4], [6], [9]–[11]. Determination of susceptibility/resistance of *M. tuberculosis* to PZA is a cumbersome and difficult task requiring the use of acidic media that renders very unreliable the interpretation of the growth readings. Therefore, PCR amplification and DNA sequencing of *pncA* is an approach of interest because it allows rapid detection of the *pncA* mutations potentially involved in PZA resistance. However, a critical point in this approach is to correctly interpret at the level of the structure/function relationships of PncA the effects of the mutations detected by the *pncA* sequencing, first because of the high degree of diversity of the mutations that can be found in PncA, and secondly, because a significant proportion of PZA-susceptible clinical isolates display amino acid substitutions in PncA that have no significant effect on the pyrazinamidase activity of the enzyme. Different models of the three-dimensional structure of PncA of *M. tuberculosis* were established with this purpose [12], [13]. The first to be reported was based on the structure of the N-carbamoylsarcosine amidohydrolase (CSHase), an enzyme harbouring, like PncA, the isochorismatase domain (PF00857) [12]. Using this model, we found that the level of activity of PncA mutants correlated with the location of the mutations with respect to the active site residue Cys138 involved in the nucleophilic attack of PZA. Du and colleagues established the crystal structure of the pyrazinamidase from *Pyrococcus horikoshii* (PhPncA) [14]. The enzyme was found to have an active cysteine residue at position 133 in the *P. horikoshii* numbering system (Cys138 in MtPncA), as well as a Zn^2+^ ion coordinated by 2 His residues, His54 and His71 (His51 and His71 in MtPncA) and one aspartate residue Asp52 (Asp49 in MtPncA). Formation of the acylenzyme was suggested to involve the thiolate form of Cys133 activated by another aspartate, Asp10 (Asp8 in MtPncA) acting as general base, and stabilized by Lys94 (Lys96 in MtPncA). A cis-peptide bond was identified between Val128-Ala129 in PhPncA (Ile133-Ala134 in MtPncA) which contributes to the formation of an oxyanion hole between the main chain NH of Cys133 on one hand and the one of Ala129 on the other (Cys138 and Ala134 in MtPncA, respectively). In PhPncA, the function of the Zn^2+^ ion was suggested to be catalytic through the activation of a water molecule for hydrolysis of the thioester bond formed between the catalytic cysteine residue and the substrate in the acylenzyme intermediate. In a more recent publication, the crystal structure of *Acinetobacter baumanii* PncA (AbPncA) in complex with nicotinic acid has been established [15]. The enzyme was found to be a divalent cation-dependent enzyme with Fe^2+^/Zn^2+^ (ratio 1∶1) hold on by 3 coordinating residues (Asp54, His56 and His89). Nicotinic acid was found to be directly tethered to the ion (assumed to be Zn^2+^ in the report), its carboxylate group being hydrogen-bounded to the main-chain amides of cis-Ala155 and Cys159 forming the oxyanion hole in AbPncA (corresponding to Ala134 and Cys138 in MtPncA).

Besides these structural studies, Zhang and colleagues characterized the PncA protein from *M. tuberculosis* at the biochemical level [16]. MtPncA was found to be a [Mn^2+^/Fe^2+^]-containing protein (ratio 1∶1). Study of mutants generated by site directed mutagenesis suggested that residues Asp8, Lys96 and Cys138 were key residues for catalysis, and Asp49, His57 and His71 for metal ion binding. Considering the structure of the pyrazinamidase from *P. horikoshii*, the only 3D-structure available at the time this study was conducted, they proposed that His57, which is naturally replaced by an aspartate residue in the PZA resistant species *Mycobacterium bovis* [4], is directly involved in metal binding, a hypothesis that was not confirmed by structural evidences.

In this report, we describe the crystal structure of the pyrazinamidase/nicotinamidase of *M. tuberculosis* at a resolution of 2.2 Å. The three-dimensional structure of the enzyme shows significant differences when compared to the structures of the pyrazinamidases from *P. horikoshii* and *A. baumanii*, mostly at the level of the loop carrying residue His57 which was observed in the *M. tuberculosis* structure to be involved in coordination of an iron ion. Comparative kinetics and thermal shift assays (TSA) of MtPncA mutants obtained by site-directed mutagenesis suggested that predicting the structure-activity relationships for PncA mutants should take into account not only the kinetics effects induced by the mutations, but also their effects on protein folding and stabilization.

## Materials and Methods

### Bacterial strains, plasmids and growth condition

Strains of *M. tuberculosis* H37Rv and *M. bovis* were from our laboratory collection. The PZA-resistant clinical strains used in this study were isolated at Pitié Salpêtrière Hospital (Paris, France). Mycobacterial DNA was extracted from colonies by heat choc treatment (1 min at 100°C, followed by 1 min at 4°C, five times). *Escherichia coli* Novablue and BL21 (DE3) competent cells (Novagen) were used as host cells for pET29a (Novagen) plasmid transformation and for expression of the PncA proteins, respectively. *E. coli* cells were grown in Luria-Bertani (LB) at 37°C in the presence of kanamycin (30 µg/ml).

### Cloning, purification and pyrazinamidase activity assay of wild-type and mutant PncA proteins

In order to purify PncA on a large scale, the *pncA* gene of strain H37Rv of *M. tuberculosis* was amplified using the amplification primers pncASCT-HindIII (^5′^TATAAGCTTTCCTCCTCAGGAGCTGCAAACCAA^3′^) (*Hind*III site underlined) and pncS-*Nde*I (^5′^GGGGAATTCCATATGCGGGCGTTGATCATC^3′^) (*Nde*I site underlined), and cloned into the *Hind*III- and *Nde*I-restricted sites of the expression vector pET29a. Point mutations were introduced by site-directed mutagenesis in the *pncA* gene cloned in the pET29a vector using the QuikChange Site-Directed Mutagenesis Kit (Stratagene).

The recombinant plasmids were introduced by transformation into *E. coli* BL21 (DE3), which was used as host cell for protein expression after induction of a 1L culture by IPTG 0.4 mM at 28°C over night. Bacterial cells were pelleted, resuspended in 20 ml of Bis-Tris 40 mM pH 6.0 DTT 1 mM, and lysed by ultrasonic treatment. The supernatant was applied onto a 2.5- x 10-cm Q Sepharose fast flow column (Pharmacia Co. Ltd., Sweden). The fractions containing PncA were eluted by a linear gradient of 0 to 1M NaCl in Bis-Tris 40 mM pH 6.0, concentrated with Amicon ultra 15 ml (Millipore) and loaded onto a gel filtration Superdex 75 (Pharmacia Co. Ltd., Sweden) previously equilibrated with Bis-Tris 40 mM pH 6.0. Finally the enzyme was concentrated onto Microcon YM30 (Millipore) to a final concentration of 20 mg/ml. The experimental molecular weight was determined by gel filtration to be 22.8 kDa. As the theoretical molecular weight of PncA is 19.6 kDa, we concluded that MtPncA is a monomeric protein.

The assay for pyrazinamidase activity was carried out as previously described [17] using 1 µM of pure enzyme and 2 mM of PZA. Specific activities were expressed in U.mg^−1^ using an extinction coefficient Δε_450_ of 6 10^−4^ µM^−1^.cm^−1^ [12].

### Thermal shift assay (TSA) of PncA proteins

TSA experiments were carried out as previously described [18] using a PTC-200 real-time PCR instrument (Bio-Rad). 50 µM of protein in BisTris 20 mM pH 6.0 was mixed with 2 µl of 5000× Sypro Orange (Molecular Probes) diluted 1∶100 in water. The samples were heated from 20 to 95°C with a heating rate of 1°C per minute. Protein thermal unfolding curves were monitored by detection of changes in fluorescence of the Sypro Orange. The melting temperature (Tm) was determined by reading the temperature at which the reaction was half- complete.

### Inductively coupled plasma-mass spectrometry (ICP MS)

Inductively coupled plasma-mass spectrometry (ICP MS) was used for metal-ion identification. Purified PncA protein was diluted to a final concentration of 5.2 mg/ml. One mL of protein solution was digested with 500 µL of HNO_3_ (69–70%) for 4 h at 85°C using a DigiPrep digestion system (SCP, Québec, Canada). Samples were analyzed by triplicate runs on a ICP MS Agilent 7500ce (Tokyo, Japan) instrument. Total Mg, Fe, Co, Mn and Zn concentrations were measured using an external calibration curve determined with reference standards for each ion.

### Crystallization and structure determination

PncA crystals were grown in sitting drops against a solution containing 1.6 M of MgSO_4_ and 0.1 M of MES at pH 6.6. Drops (2 µl) consisted in a 1∶1 (v/v) mixture of the well and protein solutions (20 mg/ml in Bis-Tris 40 mM pH 6.0). The crystals were frozen by a 100 K nitrogen gas stream without cryoprotection before data collection at the ESRF (European Synchrotron Radiation Facility, Grenoble, France, ID23-2 beamline Microfocus). The crystals belong to the hexagonal space group P6_1_22 with unit cell axes a = b = 84.1 Å and c = 100 Å, with a solvent content of 52%. The data were processed with XDS [19] and the structure was obtained by molecular replacement with the program PHASER [20] using the crystal structure of the pyrazinamidase/nicotinamidase of *Pyrococcus horikoshii* (PDB accession number 1IM5) as search model. Refinement and model building cycles were performed using CNS [21], Refmac [22], O [23] and Coot [24]. A summary of the relevant statistics of the data collection and refinement is given in Table 1. The coordinates and structure factors of PncA have been deposited in the Protein Data Bank with the PDB ID code 3PL1. Superposition between the different protein structures were performed using the secondary structure matching algorithm SSM [25] as implemented in the program Coot [24]. The figures were generated using Pymol [26].

**Table 1:** Data collection and Refinement Statistics.

Data Collection Statistics	PncA
Space group	P6_1_22
Cell dimensions	a = b = 84.1 Å, c = 100 Å
	α = β = 90°, γ = 120°
Wavelength (Å)	0.9791
Resolution range (Å) a	42.0–2.2 (2.32–2.2)
Number of unique reflections a	10481 (1260)
Redundancy a	11.6 (11.5)
Rsym (%) a	7.8 (49.8)
I/σ(I) a	20.6 (3.4)
Completeness (%)^a^	92.9 (72.5)
Refinement Statistics	
Rcrystal (%)	19.7
Rfree (%)	24
Number of proteins atoms	1372
Number of solvent atomsNumber of heterogen atom	441
r.m.s.d. bond distance (Å)	0.02
r.m.s.d. angle (°)	2.1
Average B value (Å^2^)	45

a Values in parentheses correspond to the high resolution shell.

### Molecular modeling of the PZA-PncA acyl-enzyme intermediate

The PZA structure was constructed using the module Modeler in InsightII (Accelerys). Hydrogen atoms were added and the molecule was energy minimized using CHARMM program [27] and the force field PARAM22 [28] by using the steepest-descent algorithm. The PZA-PncA acyl-enzyme intermediate structure was built using Modeler in InsightII (Accelerys). Briefly, PZA was first docked into the PncA structure using the Autodock program [29]. The best docking solution, mostly based on the value of the Autodock Intermolecular Energy parameter and the reasonableness of the interactions between the ligand and the protein, was used for the construction of the PZA-PncA acyl-enzyme intermediate which was further energy-minimized (100 steps) using the minimization module of CNS [21].

## Results

### Crystal structure of the *M. tuberculosis* PncA protein

The crystal structure of PncA was determined to 2.2 Å resolution by molecular replacement using PhPncA as search model. The asymmetric unit contained a single protein molecule. The final structure of MtPncA included 185 residues and was characterized by R_crystal_ and R_free_ of 19.7% and 24%, respectively (for details, see Table 1). Only the last C-terminal residue, Ser186, was not modeled because of the absence of electron density.

**Figure 1:**
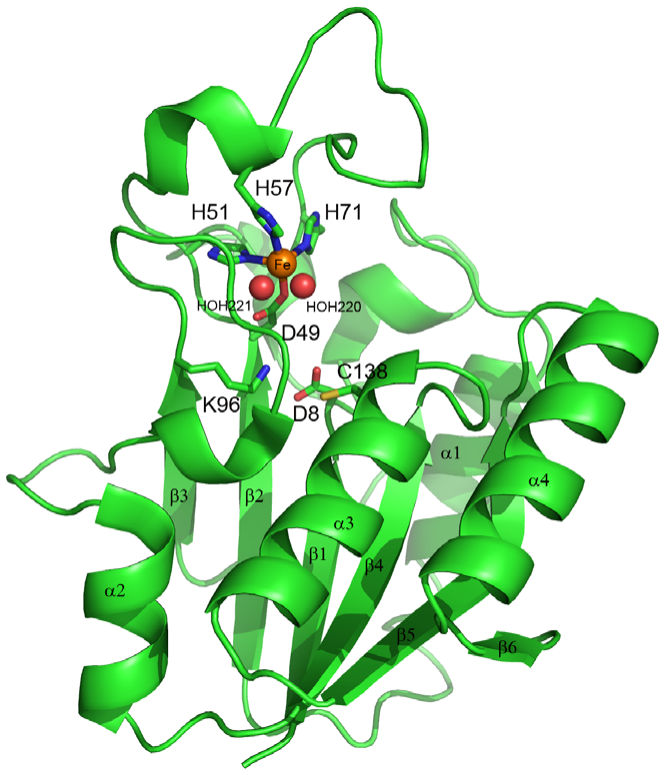
Ribbon representation of the structure of the *M. tuberculosis* PncA protein. The secondary structures, the iron binding site (Asp49, His51, His57, His71) and the catalytic triad (Cys138, Asp8, Lys96) are annotated. The iron ion is represented by the orange sphere, the two water molecules by red spheres.

The structure of PncA is made of a six-stranded parallel beta-sheet with helices packed on either side to form an α/β single domain (Figure 1). Helix α3 bears in its N-terminal part the active site cysteine residue Cys138. A strong peak was observed in the Fo-Fc map at the level of residues His51, His57, His71 and Asp49. Based on the previous report of Zhang et al. [16], we attributed in a first attempt the couple Mn^2+^/Fe^2+^ to this density, with an occupation factor of 50% for each ion. After refinement, the final B factors for Mn^2+^/Fe^2+^ were 30/34 Å^2^, respectively, which are values similar to those of the neighboring atoms. The metal ion present in PncA was finally identified by ICP-MS analysis, showing that MtPncA contained iron at a concentration of 180 µM for a total protein concentration of 260 µM in the assay. Low concentrations of zinc and manganese (42 µM and 16 µM, respectively, for a total protein concentration of 260 µM) were also detectable. A final refinement considering Fe^2+^ as the ion present in PncA showed that the iron atom was coordinated in a distorted tetragonal bipyramidal arrangement by (i) the side chains of residues His51, His71 and two water molecules, HOH220 and 221 in equatorial position, and (ii) the side chains of Asp49 and His57 in axial position (Figure 1).

### Superposition of the PncA structures and description of the *M. tuberculosis* PncA active site

**Figure 2:**
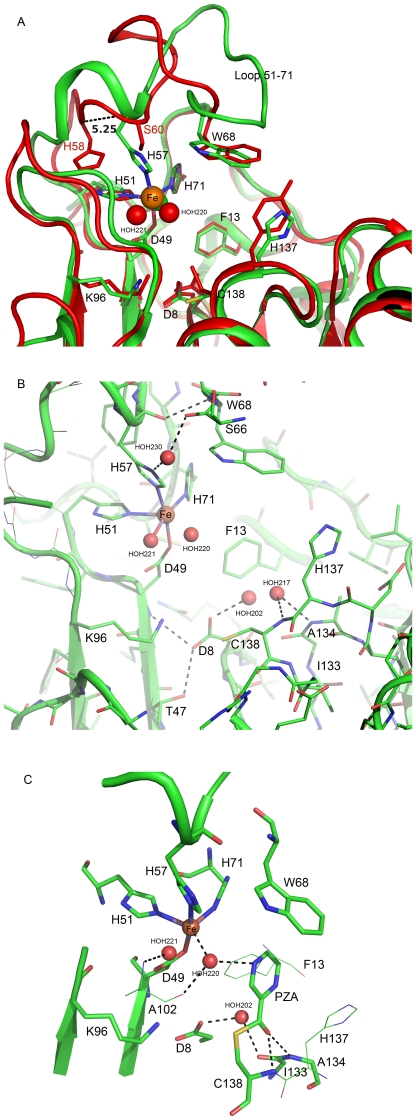
Detailed description of the active site region in MtPncA. **A** Superimposition of the active site regions in MtPncA (in green) and PhPncA (in red). The Fe2^+^ ion is depicted by an orange sphere, and water molecules by red spheres. The two proteins are shown in ribbon representation, with the side chains of interest in stick representation. **B** Details of the MtPncA active site in ribbon and stick representation. Hydrogen bonds are represented as black broken lines. **C** Modeling of the putative acyl-enzyme intermediate formed between the drug (PZA) and the side chain of Cys138 in MtPncA.

Structural alignments revealed a high level of similarity between the structure of PncA from *M. tuberculosis* presented in this report and the two crystal structures of PncA from *P. horikoshii* and *A. baumanii* (rmsds of 0.96 Å and 2.8 Å for 1IM5 and 2WT9, respectively). However, very marked structural differences were observed in two regions of PncA. One corresponded to an insertion of a stretch of 5 amino acids, VDENG, located between residues Gly108 and Thr114 in MtPncA (data not shown). The other one was localized in the loop extending from residues His51 to His71 (Figure 2A). Strikingly, this region, which constitutes the lid of the binding cavity, adopts in MtPncA a conformation displaying extensive displacements when compared to AbPncA (local rmsd of 5 Å, a large insertion of 13 amino acid residues being present in AbPncA) and PhPncA (local rmsd of 3 Å), in particular at the level of residue His57 which is located in axial position relative to the Fe ion in MtPncA, such that its side chain establishes a strong coordination bond with the metal ion (shown on Figure 2A). In the structures of PhPncA and AbPncA, the two residues occupying a positioning equivalent to that of His57 in MtPncA are Ser60 and Ser62, respectively (see Ser60 on Figure 2A). Conversely, the two His residues corresponding to His57 in the sequences of PhPncA and AbPncA (His58 and His60, respectively) are shifted by 5.2 and 4.4 Å, respectively, compared to the position of the Cα of His57 in MtPncA, such that they cannot participate in the two former structures to the binding of the metal ion (see His58 on Figure 2A).

The substrate binding cavity in MtPncA is a small crevice of approximately 10 Å deep and 7 Å wide, which is delineated on one side by a catalytic triad made of residues Lys96 at the end of strand β3, Asp8 at the end of strand β1 and Cys138 at the N-ter extremity of helice α3 (see Figures 1 and 2B). Asp8 contributes to a network of 3 H-bonds with the main chain NH of Asp49, the hydroxyl group of the side chain of Thr47 and the deacylating water molecule HOH202, respectively. Strikingly, and as opposed to previous observations made in PhPncA and AbPncA, the side chain of the key catalytic residue Cys138 in MtPncA was observed to be oriented not toward the centre of the binding cavity but, instead, toward the side chain of Lys96 (Figure 2B). On the opposite part of the active site, the 3 His residues His51, His57 and His71, together with Asp49, hold the Fe ion on, leaving a lot of room in the middle of the cavity for PZA binding. Notably, His51 and His71 correspond to the hinge of the loop encompassing residues 52 to 70 (Figure 2A). This loop, which constitutes a flap closing the binding cavity, is locked by the third coordination bond established between His57 and Fe^2+^ (Figure 2A). In the most extended part of this loop, another important residue, Trp68, is positioned above the catalytic cleft, so that it delineates, together with His137 and Phe13, the other sides of the binding site (Figure 2A). Interestingly, despite the extended rearrangements observed in the 51-71 loop in *M. tuberculosis*, Trp68 has a highly conserved positioning in PhPncA, AbPncA and MtPncA (see Figure 2A for the superimposition of MtPncA and PhPncA). In MtPncA the positioning of Trp68 is ensured by the main chain C = O of His57 establishing a direct hydrogen bonding interaction with the main chain NH of Trp68 (Figure 2B). This interaction is conserved in PhPncA and AbPncA, both enzymes displaying a similar H-bond between the main chain NH of the Trp residue and the main chain C = O of Ser60 and Ser62, respectively (data not shown). Finally, another interesting feature in the active site of PncA is the presence of a cis-peptide bond between residues Ile133 and Ala134, which orients the amide nitrogen atom of Ala134 toward the active site centre, so that it forms a potential oxyanion hole with the amide nitrogen of Cys138 (Figure 2B). In MtPncA, this potential oxyanion hole is occupied by a water molecule (HOH217).

### Molecular modeling of the acyl-enzyme complex

The structure of the acyl-enzyme complex formed between PncA and pyrazinamide has been modeled and is presented on Figure 2C. According to this model, the acylated side-chain of Cys138 clearly adopts a conformation in which the side-chain sulfur atom is reoriented toward the centre of the catalytic cleft. The carbonyl oxygen atom of PZA is ensconced in the oxyanion hole, i.e. between the NH-main chain groups of Cys138 and cis-Ala134. A water molecule, HOH202, lies immediately beneath the carbonyl carbon atom. This water molecule, which makes H-bonding interactions with the general base Asp8 on one hand, and the main chain carbonyl group of Ile133 on the other, is ideally positioned for nucleophilic attack of the acyl bond (Figure 2C). On the other side, the 6-membered ring of PZA is embedded between the three rings provided by residues Trp68, Phe13 and His137, and the positioning of the substrate is stabilized by one additional H-bond formed between the nitrogen atom N7 of the six-membered ring of PZA and the water molecule HOH220 which is itself bound to the main chain CO of Ala102 and to the Fe^2+^ ion. One can note here that the structure of the acyl-enzyme complex formed between nicotinamide and MtPncA is nearly identical to the one obtained with pyrazinamide (data not shown).

### Activities of wild-type and mutant proteins from *M. tuberculosis* and *M. bovis*, and thermal shift assay results

Eleven PncA mutants were constructed by site-directed mutagenesis, over-expressed in *E*. *coli* BL21(DE3) and purified in order to study their thermal stability and their catalytic behavior. Of the 11 mutants, one contained a phenotypically neutral substitution, Cys184Ser, introduced far from the active site residues, at the level of the penultimate residue of the polypeptide chain on the C-terminal helix α4. The 10 other mutants included His51Ala, His57Asp and Asp49Gly at the level of the Fe^2^+ binding site, Asp8Leu, Asp8Glu, Lys96Gln, Ala134Val and Cys138Ala in the catalytic site, and Phe13Leu and Trp68Leu in the PZA binding cavity.

**Figure 3:**
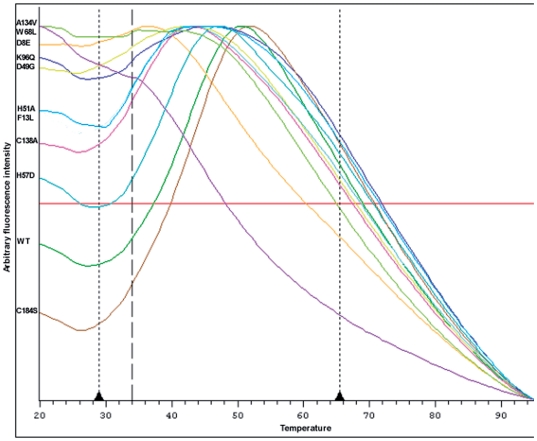
Unfolding of MtPncA and various mutants monitored in the presence of SYPRO orange. The proteins were gradually heated and the unfolding process was monitored by detection of the charges of fluorescence of the dye reporter that binds preferentially to the unfolded proteins [18]. The fluorescence intensities (Y axis) are measured according to the temperature (X axis, in °C).

As expected, the Cys184Ser mutant was produced with a yield comparable to that of the wild-type enzyme (18.5 versus 27 mg of pure protein obtained from a 1 litre culture, respectively) (Table 2). The two proteins also shared similar behavior in kinetic analyses (specific activity of 22 and 24 U.mg^−1^, respectively) and in TSA experiments (Tm  = 43°C). In both cases, binding of the Sypro-orange fluorescent probe resulted first in an increase in fluorescence intensity corresponding to the increase in dye binding due to the exposition of the buried hydrophobic regions at the beginning of the unfolding process. After a short transition by a maximum of fluorescence, this phase was followed by a drop of the fluorescent signal emitted by the probe due to global destructuring of the protein when temperature reaches too high values (see Figure 3).

**Table 2:** Biochemical properties of wild-type and mutant PncA proteins.

PncA proteins	Yield of protein (mg)	TSA resultsIFI a	Tm b	Specificactivity c
WtC184SD8LD8EK96QA134VC138AD49G	2718.5pp d1.46.21.86.81.9	10.52.32.22.51.52.2	43433235nd e3833	24220.02000.01000
H51AH57DW68LF13L	2.72.48.417	1.91.52.51.9	3539nd e35	0.250.0050.0050.02

a IFI, initial fluorescence intensity, expressed comparatively to that of the wild-type enzyme set to 1.

b Tm in °C.

c Specific activities expressed in U.mg^−1^.

d pp, not determinable because of protein precipitation.

e nd, not determinable.

By contrast, most of the other PncA mutants were found to be produced at yields up to 19-fold lower than those observed for the wild-type Pnca protein, the mutant Asp8Leu being completely lost during the course of the purification process because of protein precipitation (Table 2). As Asp8Leu precipitated, we constructed another mutant, Asp8Glu, which was produced at a low yield (1.4 mg of pure protein per liter of culture). Interestingly, the Asp8Glu mutation drastically modified the nature of the unfolding curve obtained by TSA. Indeed, this mutant showed a relatively high level of fluorescence at low temperatures, i.e. in the 20–30°C range preceding the initial unfolding phase (Figure 3). Moreover, the Tm value of the mutant (32°C) was decreased by 11°C when compared to the wild-type enzyme, and the mutant was characterized in the kinetic assays by a drastic decrease of PZase activity (0.020 U/mg) (Table 2).

The other mutations introduced in the active site (Lys96Gln, Ala134Val, Cys138Ala, Asp49Gly, His51Ala, His57Asp, Trp68Leu, and Phe13Leu) had very similar effects. Most of the mutants, except Phe13Leu, showed a marked reduction in the purification yields (1.8 to 8.4 mg per litre of culture, with an average value of 4.3 mg per litre of culture). On TSA analysis, they displayed atypical unfolding curves characterized by high fluorescence intensities at low temperature (increased by 1.5 to 2.5 fold) (see Figure 3 and Table 2) and Tm values (33 to 39°C) significantly decreased compared to the wild-type enzyme (43°C) (Table 2). Mutants harboring the amino acid changes Trp68Leu, Ala134Val, Asp8Glu, Lys96Gln and Asp49Gly displayed the most marked effects, in particular Trp68Leu for which the initial unfolding transition was hardly detectable as the protein underwent significant unfolding between 30–40°C, which is the range of temperature usually corresponding to the transition during which fluorescence raises for the other mutants (Figure 3). For Ala134Val, the initial unfolding phase was too flat to allow determination of the midpoint of the unfolding phase. As indicated by the values of their specific activities, all the mutants were affected in the PZase activity, with no detectable PZA hydrolysis for Cys138Ala, Lys96Gln and Asp49Gly, and a very low residual activity for Ala134Val, His51Ala, His57Asp, Trp68Leu and Phe13Leu (Table 2).

## Discussion

The overall architecture of the pyrazinamidase of *M. tuberculosis* is similar to that reported for the other pyrazinamidases of *A. baumanii* and *P. horikoshii*. However, several striking structural variations that have been specifically detected in the crystal structure of MtPncA, are of particular note. First, analysis of the metal ion content of MtPncA by ICP-MS analysis indicated that the enzyme preferentially contains iron. Thus, our results are rather in agreement with the previous report of Zhang et al. [16] who found that the *M. tuberculosis* pyrazinamidase contains iron and manganese with a molar ratio 1∶1, as opposed to what was speculated from the study of the pyrazinamidase of *P. horikoshii* which hypothesized that the metal ion present in PncA is a zinc playing a direct catalytic role by promoting a hydroxyl ion involved in hydrolysis of the drug [14]. The latter assumption seems to be definitively ruled out considering the fact that the metal ion in MtPncA is located too far from the active site cysteine residue (Cys138) to be capable to contribute directly to PZA hydrolysis through the activation of a water molecule. However, it cannot be excluded that manganese or zinc can also occupy the metal binding site in MtPncA, as recently suggested in a study that identified the presence of manganese in MtPncA by EPR spectroscopy [30] and as observed in the present study showing the presence of low amounts of zinc and manganese in MtPncA.

The second striking structural feature observed in MtPncA corresponds to the very peculiar conformation of the 51–71 loop forming a protrusion that occludes the mouth of the binding cavity. Interestingly, the most significant structural variations observed between MtPncA on one hand and PhPncA and AbPncA on the other, are concentrated in this part of the protein. One of the most crucial structural elements in this loop appears to be the specific positioning of residue His57 which is directly involved in the coordination of the Fe^2+^ ion. Thus, this residue undoubtedly constitutes an essential element of the scaffolding of the loop by ensuring a covalent stabilization at the level of residue 57 which, itself, is involved in the positioning of Trp68 (direct H-bonding interaction between the main chain C = O of His57 and the main chain NH of Trp68) and of Ser66 (indirect interaction with the side chain of His57 via the water molecule HOH230) (see Figure 2B). The structural bases for the specific conformation of loop 51–71 at the level of His57 in *M. tuberculosis*, which is not involved in protein-protein interactions in the crystal packing, are likely related to the specific amino acid sequence found in the 51–71 region around His57. Notably, in PhPncA and AbPncA, the motif in this region is D_49_WH_51_PXXH_55_, (where X represents a non-conserved residue) whereas in MtPncA the sequence is D_49_FH_51_XXPXXH_57_, such that an insertion of 2 additional residues is specifically observed in MtPncA between His51 and the PXXH_57_ motif. This insertion in MtPncA allows His57 to be shifted toward the metal binding site and correctly positioned to participate in the coordination of the Fe^2+^ ion. Taken altogether, these observations readily account for the fact that *M. bovis*, which possesses a histidine to aspartate substitution in position 57, produces an inactive PncA protein and is, consequently, naturally resistant to PZA [4].

On the basis of the MtPncA structure reported here, the addition-elimination mechanism previously proposed for the pyrazinamidase of *A. baumanii* [15] likely applies in *M. tuberculosis*. According to this mechanism, the binding of the drug would be mostly ensured by (i) the hydrogen bonding interaction formed between the pyridyl nitrogen atom of PZA and the water molecule HOH220 coordinated to the Fe^2+^ ion and (ii) the strong interaction of the carbonyl-oxygen atom of PZA in the Ala134-Cys138 oxyanion hole. In a second stage, the nucleophilic thiolate form of Cys138, promoted by Asp8, would attack the carbonyl carbon atom of PZA, resulting in the release of ammonia and formation of the acyl-enzyme intermediate shown on Figure 2C. The latter compound would be finally hydrolysed by the water molecule HOH202 ideally positioned and activated by the general base Asp8 to perform the final hydrolytic step.

A major new finding in the study of the PncA mutants presented in this report is the essential contribution of the studied residues not only to catalysis but also to protein folding and thermal stability. For all the studied mutants (excluding Cys184Ser which behaved like the wild-type enzyme), total loss or very low residual levels of PZase activity was observed whatever was their contribution in MtPncA (catalytic triad for Cys138Ala, Asp8Glu and Lys96Gln, formation of the oxyanion hole for Ala134Val, Fe coordination for Asp49Gly, His51Ala and His57Asp, substrate binding for Trp68Leu and Phe13Leu), a result that confirms their importance in the PZase activity of MtPncA. The results of the TSA assay indicated that most of the mutants also exhibited drastic changes in their unfolding curves compared to the wild-type MtPncA protein, most particularly Asp8Glu, Lys96Gln, Ala134Val, Asp49Gly and Trp68Leu (Figure 3). The major effect induced by these mutations corresponded to a marked increase in the initial fluorescence values and, for the two mutants Ala134Val and Trp68Leu, to the absence of unfolding transition at the beginning of heating (30–40°C). These observations are consistent with the hypothesis that such mutants would display a significant degree of unfolding at 20°C, at least in the region of the binding site, which would increase the accessibility of the fluorescent reporter to hydrophobic regions of the protein prior to thermal treatment, as suggested in a recent report applying the thermal shift assay to the investigation of mitotic kinesins [31]. In addition, these mutants exhibited significant decrease in thermal stability, as reflected by Tm values not determinable for Ala134Val and Trp68Leu, and decreased by 8 to 11°C for Asp8Glu, Asp49Gly and Lys96Gln, corroborating the idea that these mutations are associated with significant effects on the integrity of the 3D structure of PncA. Regarding the 4 remaining mutants, His51Ala, His57Asp, Cys138Ala and Phe13Leu, their consequences on unfolding curves and Tm shifts were less pronounced, suggesting that these mutations have more limited effects on folding and stability of MtPncA.

In conclusion, the crystal structure of the pyrazinamidase from *M. tuberculosis* described here has unveiled important structural features of the enzyme and so, represents a valuable tool to decipher the structure-function relationships and investigate the molecular mechanisms of resistance to PZA stemming from the point mutations identified in clinical isolates. In particular, it highlights the important role played by the Fe^2+^ ion and His57 for local stabilisation of the 51–71 loop, accounting for the production of defective PncA protein in *M. bovis* which naturally possesses an aspartate at this position. Finally, it highlights the underestimated importance of protein folding and thermal stability in PZase activity of the PncA mutants produced in clinical isolates, a parameter which has to be taken into account to fully understand resistance to PZA.

## References

[1] Zhang Y, Mitchison D (2003). The curious characteristics of pyrazinamide: a review.. Int J Tuberc Lung Dis.

[2] Konno K, Feldmann FM, McDermott W (1967). Pyrazinamide susceptibility and amidase activity of tubercle bacilli.. Am Rev Respir Dis.

[3] Scorpio A, Zhang Y (1996). Mutations in *pncA*, a gene encoding pyrazinamidase/nicotinamidase, cause resistance to the antituberculous drug pyrazinamide in tubercle bacillus.. Nat Med.

[4] Scorpio A, Collins D, Whipple D, Cave D, Bates J (1997). Rapid differentiation of bovine and human tubercle bacilli based on a characteristic mutation in the bovine pyrazinamidase gene.. J Clin Microbiol.

[5] Doustdar F, Khosravi AD, Farnia P (2009). *Mycobacterium tuberculosis* genotypic diversity in pyrazinamide-resistant isolates of Iran.. Microb Drug Resist.

[6] Lemaitre N, Sougakoff W, Truffot-Pernot C, Jarlier V (1999). Characterization of new mutations in pyrazinamide-resistant strains of *Mycobacterium tuberculosis* and identification of conserved regions important for the catalytic activity of the pyrazinamidase PncA.. Antimicrob Agents Chemother.

[7] Pandey S, Newton S, Upton A, Roberts S, Drinkovic D (2009). Characterisation of pncA mutations in clinical *Mycobacterium tuberculosis* isolates in New Zealand.. Pathology.

[8] Zhang H, Bi LJ, Li CY, Sun ZG, Deng JY (2009). Mutations found in the pncA gene of *Mycobacterium tuberculosis* in clinical pyrazinamide-resistant isolates from a local region of China.. J Int Med Res.

[9] Jureen P, Werngren J, Toro JC, Hoffner S (2008). Pyrazinamide resistance and *pncA* gene mutations in *Mycobacterium tuberculosis*.. Antimicrob Agents Chemother.

[10] Sekiguchi J, Nakamura T, Miyoshi-Akiyama T, Kirikae F, Kobayashi I (2007). Development and evaluation of a line probe assay for rapid identification of pncA mutations in pyrazinamide-resistant mycobacterium tuberculosis strains.. J Clin Microbiol.

[11] Zimic M, Sheen P, Quiliano M, Gutierrez A, Gilman RH (2010). Peruvian and globally reported amino acid substitutions on the *Mycobacterium tuberculosis* pyrazinamidase suggest a conserved pattern of mutations associated to pyrazinamide resistance.. Infect Genet Evol.

[12] Lemaitre N, Callebaut I, Frenois F, Jarlier V, Sougakoff W (2001). Study of the structure-activity relationships for the pyrazinamidase (PncA) from *Mycobacterium tuberculosis*.. Biochem J.

[13] Unissa A, Selvakumar N, Hassan S (2010). Insight to pyrazinamide resistance in *Mycobacterium tuberculosis* by molecular docking.. Bioinformation.

[14] Du X, Wang W, Kim R, Yakota H, Nguyen H (2001). Crystal structure and mechanism of catalysis of a pyrazinamidase from *Pyrococcus horikoshii*.. Biochemistry.

[15] Fyfe PK, Rao VA, Zemla A, Cameron S, Hunter WN (2009). Specificity and mechanism of *Acinetobacter baumanii* nicotinamidase: implications for activation of the front-line tuberculosis drug pyrazinamide.. Angew Chem Int Ed Engl.

[16] Zhang H, Deng JY, Bi LJ, Zhou YF, Zhang ZP (2008). Characterization of *Mycobacterium tuberculosis* nicotinamidase/pyrazinamidase.. FEBS J.

[17] Sheen P, Ferrer P, Gilman RH, Lopez-Llano J, Fuentes P (2009). Effect of pyrazinamidase activity on pyrazinamide resistance in *Mycobacterium tuberculosis*.. Tuberculosis (Edinb).

[18] Pantoliano MW, Petrella EC, Kwasnoski JD, Lobanov VS, Myslik J (2001). High-density miniaturized thermal shift assays as a general strategy for drug discovery.. J Biomol Screen.

[19] Kabsch W (1993). Automatic processing of rotation diffraction data from crystals of initially unknown symmetry and cell constants.. Journal of Applied Crystallography.

[20] McCoy AJ, Grosse-Kunstleve RW, Adams PD, Winn MD, Storoni LC (2007). Phaser crystallographic software.. Journal of Applied Crystallography.

[21] Brunger AT, Adams PD, Clore GM, Delano WL, Gros P (1998). Crystallography and NMR system: a new software suite for macromolecular structure determination.. Acta Crystallography D.

[22] Vagin AA, Steiner RA, Lebedev AA, Potterton L, McNicholas S (2004). REFMAC5 dictionary: organization of prior chemical knowledge and guidelines for its use.. Acta Crystallography D.

[23] Jones TA, Zou JY, Cowan SW, Kjeldgaard M (1991). Improved methods for building protein models in electron density maps and the location of errors in these models.. Acta Crystallogr A.

[24] Emsley P, Cowtan K (2004). Coot: model-building tools for molecular graphics.. Acta Crystallogr D Biol Crystallogr.

[25] Krissinel E, Henrick K (2004). Secondary-structure matching (SSM), a new tool for fast protein structure alignment in three dimensions.. Acta Crystallogr D Biol Crystallogr.

[26] DeLano WL (2002). The Pymol Molecular Graphics System.. DeLano Scientific.

[27] Brooks BR, Bruccoleri RE, Olafson BD, States DJ, Swaminathan S (1983). CHARMM: a program for macromolecular energy minimization and dynamics calculations.. Journal of Computational Chemistry.

[28] MacKerell AD, Bashford D, Bellot M, Dunbrack RL, Evanseck JD (1998). All-atom empirical potential for molecular modelling and dynamics studies of proteins.. Journal of Physical Chemistry.

[29] Morris GM, Goodsell DS, Halliday RS, Huey R, Hart WE, Belew RK, Olson AJ (1998). Automated docking using a Lamarckian genetic algorithm and empirical binding free energy function.. Journal of Computational Chemistry.

[30] Seiner DR, Hegde SS, Blanchard JS (2010). Kinetics and Inhibition of Nicotinamidase from *Mycobacterium tuberculosis*.. Biochemistry.

[31] McDonnell PA, Yanchunas J, Newitt JA, Tao L, Kiefer SE (2009). Assessing compound binding to the Eg5 motor domain using a thermal shift assay.. Anal Biochem.

